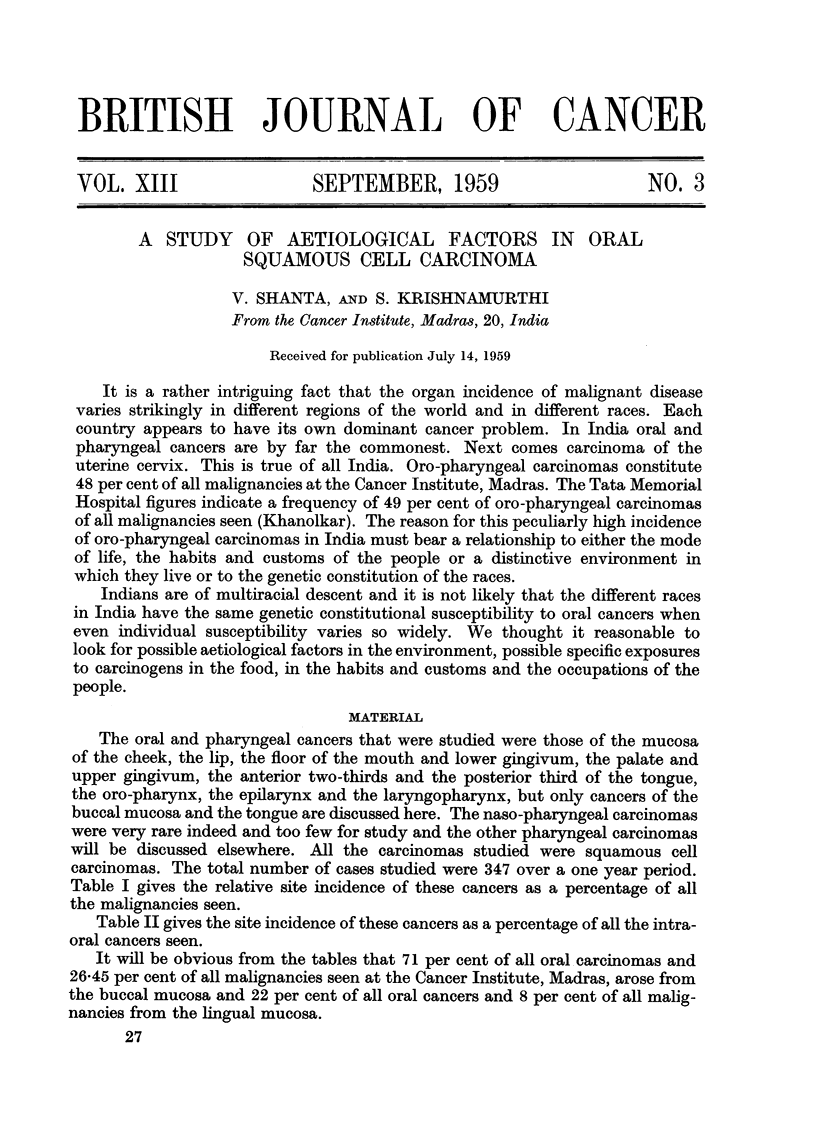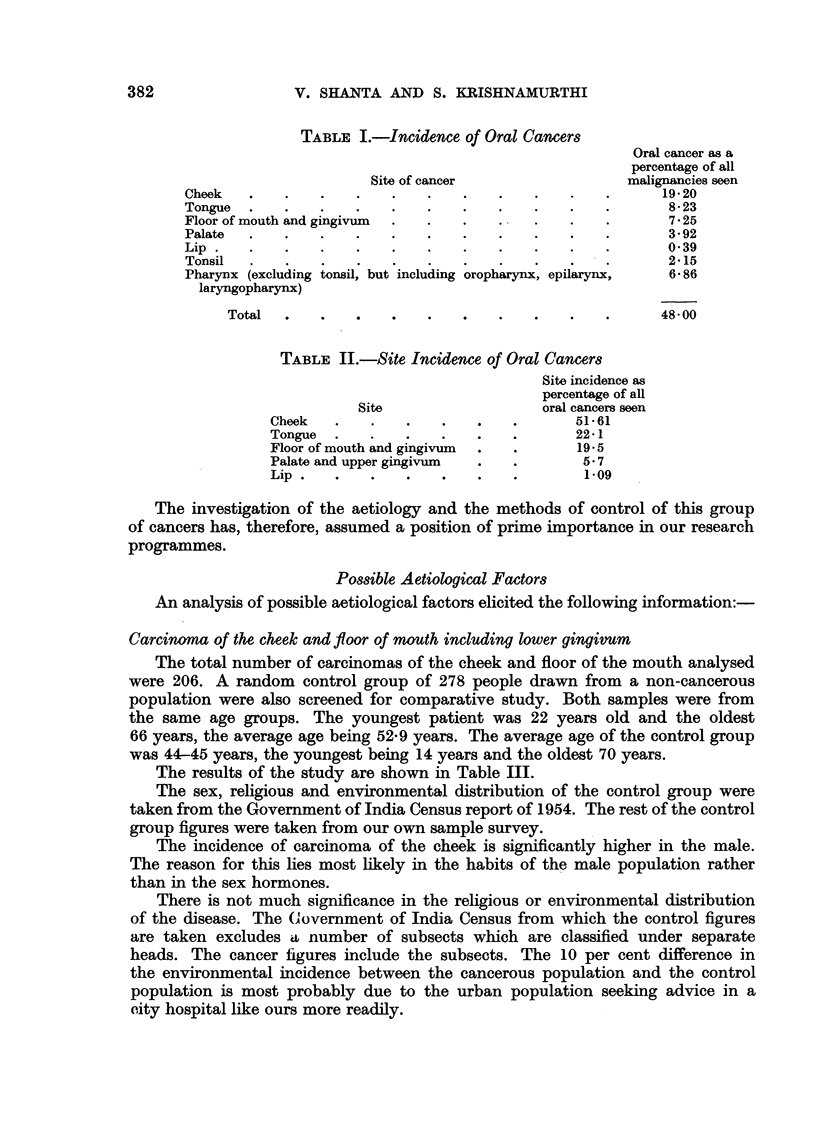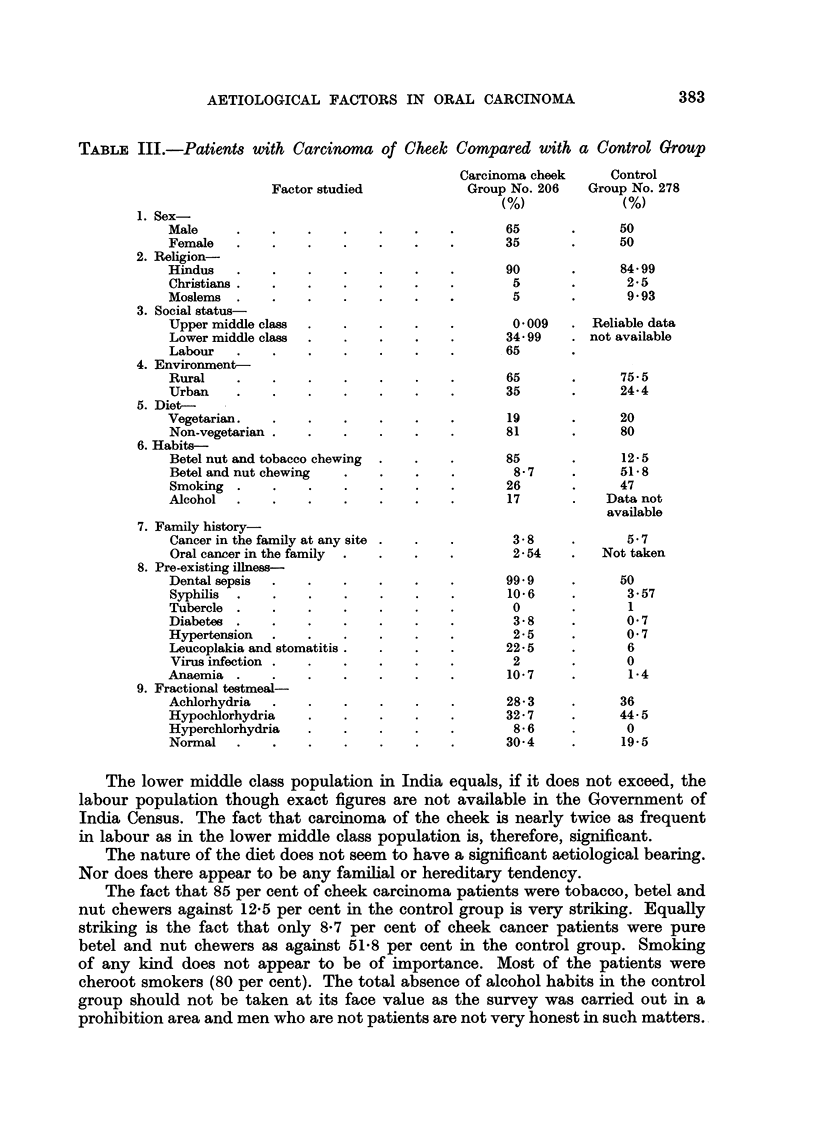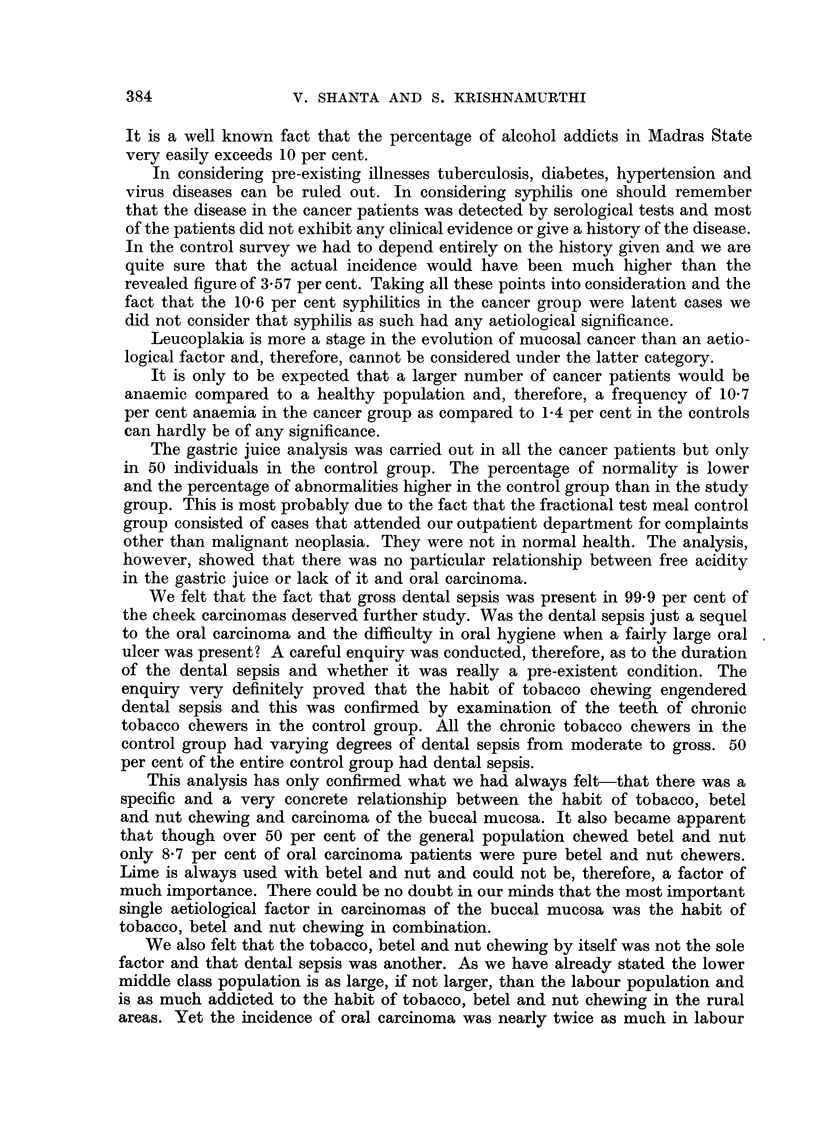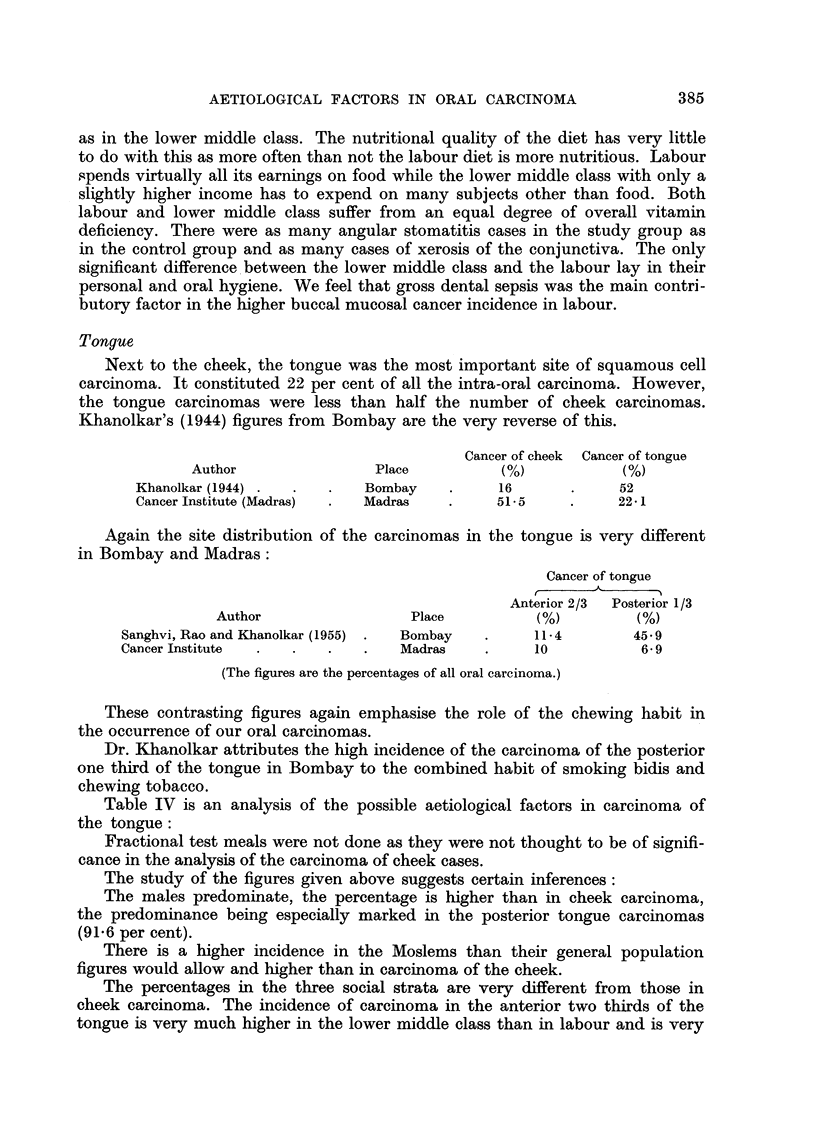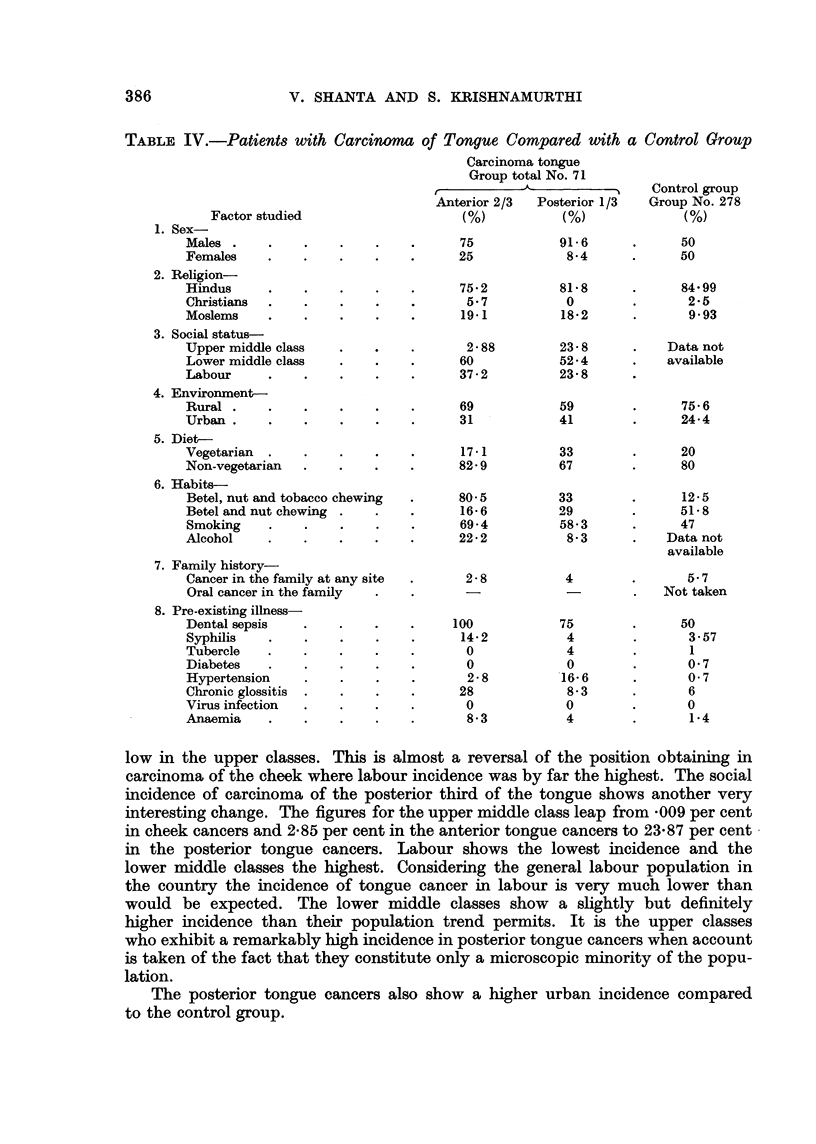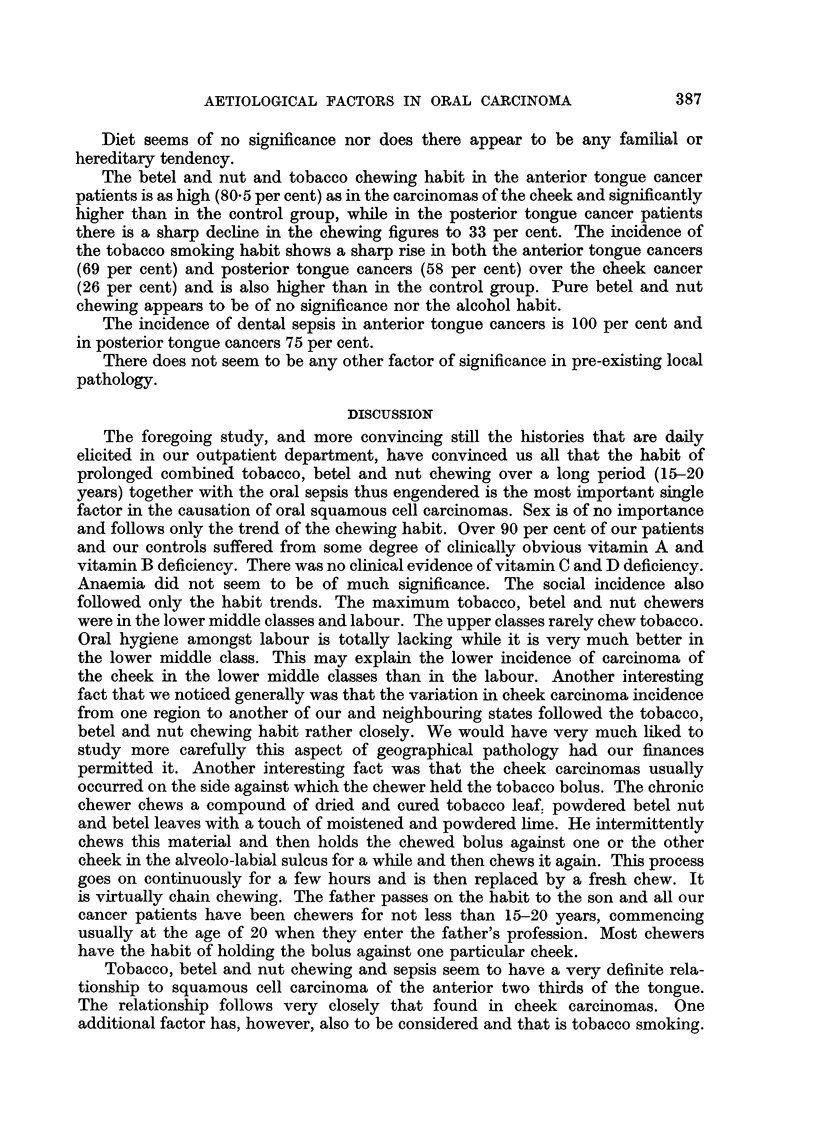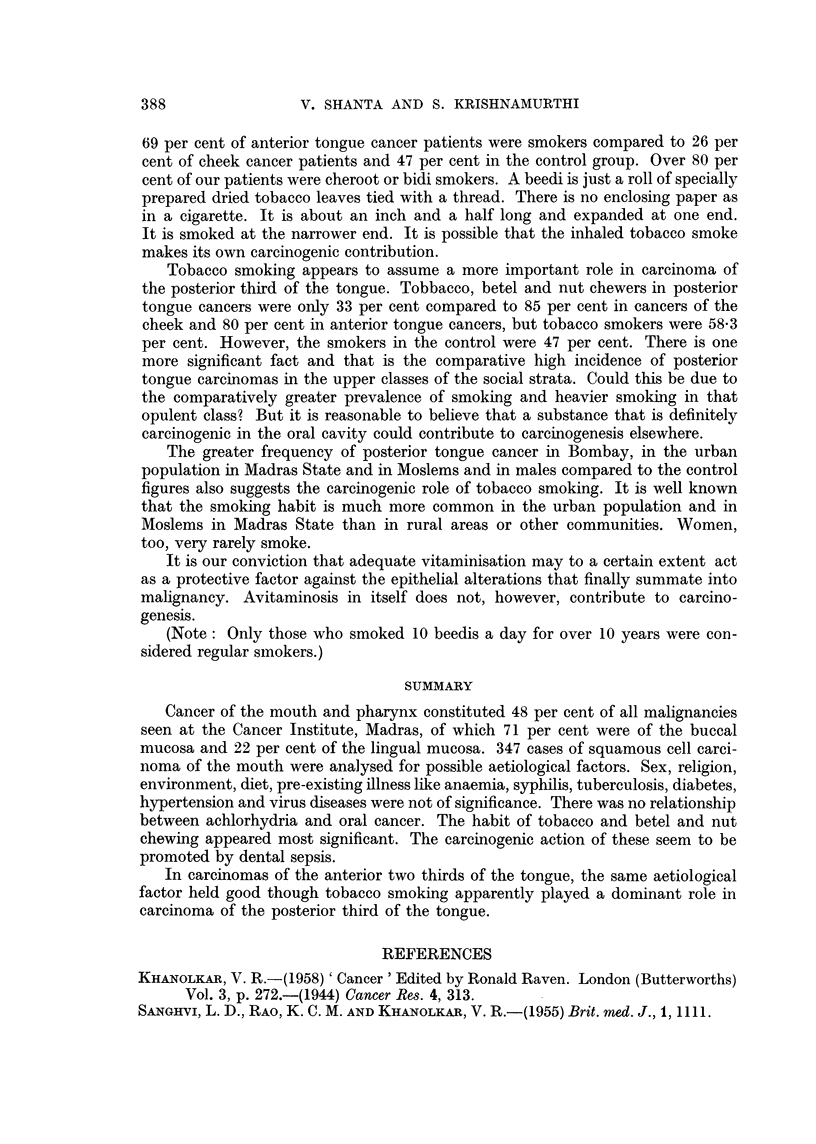# A Study of Aetiological Factors in Oral Squamous Cell Carcinoma

**DOI:** 10.1038/bjc.1959.43

**Published:** 1959-09

**Authors:** V. Shanta, S. Krishnamurthi


BRITISH JOURNAL OF CANCER

VOL. XIII SEPTEMBER, 1959 NO. 3

### A STUDY OF AETIOLOGICAL FACTORS IN ORAL SQUAMOUS CELL CARCINOMA

V. SHANTA, AND S. KRISHNAMURTHI

*From the Cancer Institute, Madras, 20, India*

Received for publication July 14, 1959

It is a rather intriguing fact that the organ incidence of malignant disease varies strikingly in different regions of the world and in different races. Each country appears to have its own dominant cancer problem. In India oral and pharyngeal cancers are by far the commonest. Next comes carcinoma of the uterine cervix. This is true of all India. Oro-pharyngeal carcinomas constitute 48 per cent of all malignancies at the Cancer Institute, Madras. The Tata Memorial Hospital figures indicate a frequency of 49 per cent of oro-pharyngeal carcinomas of all malignancies seen (Khanolkar). The reason for this peculiarly high incidence of oro-pharyngeal carcinomas in India must bear a relationship to either the mode of life, the habits and customs of the people or a distinctive environment in which they live or to the genetic constitution of the races.

Indians are of multiracial descent and it is not likely that the different races in India have the same genetic constitutional susceptibility to oral cancers when even individual susceptibility varies so widely. We thought it reasonable to look for possible aetiological factors in the environment, possible specific exposures to carcinogens in the food, in the habits and customs and the occupations of the people.

### MATERIAL

The oral and pharyngeal cancers that were studied were those of the mucosa of the cheek, the lip, the floor of the mouth and lower gingivum, the palate and upper gingivum, the anterior two-thirds and the posterior third of the tongue, the oro-pharynx, the epilarynx and the laryngopharynx, but only cancers of the buccal mucosa and the tongue are discussed here. The naso-pharyngeal carcinomas were very rare indeed and too few for study and the other pharyngeal carcinomas will be discussed elsewhere. All the carcinomas studied were squamous cell carcinomas. The total number of cases studied were 347 over a one year period. Table I gives the relative site incidence of these cancers as a percentage of all the malignancies seen.

Table II gives the site incidence of these cancers as a percentage of all the intra-oral cancers seen.

It will be obvious from the tables that 71 per cent of all oral carcinomas and 26·45 per cent of all malignancies seen at the Cancer Institute, Madras, arose from the buccal mucosa and 22 per cent of all oral cancers and 8 per cent of all malignancies from the lingual mucosa.

TABLE I.—*Incidence of Oral Cancers*

| Site of cancer | Oral cancer as a percentage of all malignancies seen |
|---|---|
| Cheek | 19·20 |
| Tongue | 8·23 |
| Floor of mouth and gingivum | 7·25 |
| Palate | 3·92 |
| Lip | 0·39 |
| Tonsil | 2·15 |
| Pharynx (excluding tonsil, but including oropharynx, epilarynx, laryngopharynx) | 6·86 |
| Total | 48·00 |

TABLE II.—*Site Incidence of Oral Cancers*

| Site | Site incidence as percentage of all oral cancers seen |
|---|---|
| Cheek | 51·61 |
| Tongue | 22·1 |
| Floor of mouth and gingivum | 19·5 |
| Palate and upper gingivum | 5·7 |
| Lip | 1·09 |

The investigation of the aetiology and the methods of control of this group of cancers has, therefore, assumed a position of prime importance in our research programmes.

*Possible Aetiological Factors*

An analysis of possible aetiological factors elicited the following information:—

*Carcinoma of the cheek and floor of mouth including lower gingivum*

The total number of carcinomas of the cheek and floor of the mouth analysed were 206. A random control group of 278 people drawn from a non-cancerous population were also screened for comparative study. Both samples were from the same age groups. The youngest patient was 22 years old and the oldest 66 years, the average age being 52·9 years. The average age of the control group was 44–45 years, the youngest being 14 years and the oldest 70 years.

The results of the study are shown in Table III.

The sex, religious and environmental distribution of the control group were taken from the Government of India Census report of 1954. The rest of the control group figures were taken from our own sample survey.

The incidence of carcinoma of the cheek is significantly higher in the male. The reason for this lies most likely in the habits of the male population rather than in the sex hormones.

There is not much significance in the religious or environmental distribution of the disease. The Government of India Census from which the control figures are taken excludes a number of subsects which are classified under separate heads. The cancer figures include the subsects. The 10 per cent difference in the environmental incidence between the cancerous population and the control population is most probably due to the urban population seeking advice in a city hospital like ours more readily.

TABLE III.—*Patients with Carcinoma of Cheek Compared with a Control Group*

| Factor studied | Carcinoma cheek Group No. 206 (%) | Control Group No. 278 (%) |
|---|---|---|
| 1. Sex— | | |
| Male | 65 | 50 |
| Female | 35 | 50 |
| 2. Religion— | | |
| Hindus | 90 | 84·99 |
| Christians | 5 | 2·5 |
| Moslems | 5 | 9·93 |
| 3. Social status— | | |
| Upper middle class | 0·009 | Reliable data |
| Lower middle class | 34·99 | not available |
| Labour | 65 | |
| 4. Environment— | | |
| Rural | 65 | 75·5 |
| Urban | 35 | 24·4 |
| 5. Diet— | | |
| Vegetarian | 19 | 20 |
| Non-vegetarian | 81 | 80 |
| 6. Habits— | | |
| Betel nut and tobacco chewing | 85 | 12·5 |
| Betel and nut chewing | 8·7 | 51·8 |
| Smoking | 26 | 47 |
| Alcohol | 17 | Data not available |
| 7. Family history— | | |
| Cancer in the family at any site | 3·8 | 5·7 |
| Oral cancer in the family | 2·54 | Not taken |
| 8. Pre-existing illness— | | |
| Dental sepsis | 99·9 | 50 |
| Syphilis | 10·6 | 3·57 |
| Tubercle | 0 | 1 |
| Diabetes | 3·8 | 0·7 |
| Hypertension | 2·5 | 0·7 |
| Leucoplakia and stomatitis | 22·5 | 6 |
| Virus infection | 2 | 0 |
| Anaemia | 10·7 | 1·4 |
| 9. Fractional testmeal— | | |
| Achlorhydria | 28·3 | 36 |
| Hypochlorhydria | 32·7 | 44·5 |
| Hyperchlorhydria | 8·6 | 0 |
| Normal | 30·4 | 19·5 |

The lower middle class population in India equals, if it does not exceed, the labour population though exact figures are not available in the Government of India Census. The fact that carcinoma of the cheek is nearly twice as frequent in labour as in the lower middle class population is, therefore, significant.

The nature of the diet does not seem to have a significant aetiological bearing. Nor does there appear to be any familial or hereditary tendency.

The fact that 85 per cent of cheek carcinoma patients were tobacco, betel and nut chewers against 12·5 per cent in the control group is very striking. Equally striking is the fact that only 8·7 per cent of cheek cancer patients were pure betel and nut chewers as against 51·8 per cent in the control group. Smoking of any kind does not appear to be of importance. Most of the patients were cheroot smokers (80 per cent). The total absence of alcohol habits in the control group should not be taken at its face value as the survey was carried out in a prohibition area and men who are not patients are not very honest in such matters.

It is a well known fact that the percentage of alcohol addicts in Madras State very easily exceeds 10 per cent.

In considering pre-existing illnesses tuberculosis, diabetes, hypertension and virus diseases can be ruled out. In considering syphilis one should remember that the disease in the cancer patients was detected by serological tests and most of the patients did not exhibit any clinical evidence or give a history of the disease. In the control survey we had to depend entirely on the history given and we are quite sure that the actual incidence would have been much higher than the revealed figure of 3·57 per cent. Taking all these points into consideration and the fact that the 10·6 per cent syphilitics in the cancer group were latent cases we did not consider that syphilis as such had any aetiological significance.

Leucoplakia is more a stage in the evolution of mucosal cancer than an aetiological factor and, therefore, cannot be considered under the latter category.

It is only to be expected that a larger number of cancer patients would be anaemic compared to a healthy population and, therefore, a frequency of 10·7 per cent anaemia in the cancer group as compared to 1·4 per cent in the controls can hardly be of any significance.

The gastric juice analysis was carried out in all the cancer patients but only in 50 individuals in the control group. The percentage of normality is lower and the percentage of abnormalities higher in the control group than in the study group. This is most probably due to the fact that the fractional test meal control group consisted of cases that attended our outpatient department for complaints other than malignant neoplasia. They were not in normal health. The analysis, however, showed that there was no particular relationship between free acidity in the gastric juice or lack of it and oral carcinoma.

We felt that the fact that gross dental sepsis was present in 99·9 per cent of the cheek carcinomas deserved further study. Was the dental sepsis just a sequel to the oral carcinoma and the difficulty in oral hygiene when a fairly large oral ulcer was present? A careful enquiry was conducted, therefore, as to the duration of the dental sepsis and whether it was really a pre-existent condition. The enquiry very definitely proved that the habit of tobacco chewing engendered dental sepsis and this was confirmed by examination of the teeth of chronic tobacco chewers in the control group. All the chronic tobacco chewers in the control group had varying degrees of dental sepsis from moderate to gross. 50 per cent of the entire control group had dental sepsis.

This analysis has only confirmed what we had always felt—that there was a specific and a very concrete relationship between the habit of tobacco, betel and nut chewing and carcinoma of the buccal mucosa. It also became apparent that though over 50 per cent of the general population chewed betel and nut only 8·7 per cent of oral carcinoma patients were pure betel and nut chewers. Lime is always used with betel and nut and could not be, therefore, a factor of much importance. There could be no doubt in our minds that the most important single aetiological factor in carcinomas of the buccal mucosa was the habit of tobacco, betel and nut chewing in combination.

We also felt that the tobacco, betel and nut chewing by itself was not the sole factor and that dental sepsis was another. As we have already stated the lower middle class population is as large, if not larger, than the labour population and is as much addicted to the habit of tobacco, betel and nut chewing in the rural areas. Yet the incidence of oral carcinoma was nearly twice as much in labour as in the lower middle class. The nutritional quality of the diet has very little to do with this as more often than not the labour diet is more nutritious. Labour spends virtually all its earnings on food while the lower middle class with only a slightly higher income has to expend on many subjects other than food. Both labour and lower middle class suffer from an equal degree of overall vitamin deficiency. There were as many angular stomatitis cases in the study group as in the control group and as many cases of xerosis of the conjunctiva. The only significant difference between the lower middle class and the labour lay in their personal and oral hygiene. We feel that gross dental sepsis was the main contributory factor in the higher buccal mucosal cancer incidence in labour.

*Tongue*

Next to the cheek, the tongue was the most important site of squamous cell carcinoma. It constituted 22 per cent of all the intra-oral carcinoma. However, the tongue carcinomas were less than half the number of cheek carcinomas. Khanolkar's (1944) figures from Bombay are the very reverse of this.

| Author | Place | Cancer of cheek (%) | Cancer of tongue (%) |
|---|---|---|---|
| Khanolkar (1944) | Bombay | 16 | 52 |
| Cancer Institute (Madras) | Madras | 51·5 | 22·1 |

Again the site distribution of the carcinomas in the tongue is very different in Bombay and Madras :

| Author | Place | Cancer of tongue | |
|---|---|---|---|
| | | Anterior 2/3 (%) | Posterior 1/3 (%) |
| Sanghvi, Rao and Khanolkar (1955) | Bombay | 11·4 | 45·9 |
| Cancer Institute | Madras | 10 | 6·9 |

(The figures are the percentages of all oral carcinoma.)

These contrasting figures again emphasise the role of the chewing habit in the occurrence of our oral carcinomas.

Dr. Khanolkar attributes the high incidence of the carcinoma of the posterior one third of the tongue in Bombay to the combined habit of smoking bidis and chewing tobacco.

Table IV is an analysis of the possible aetiological factors in carcinoma of the tongue :

Fractional test meals were not done as they were not thought to be of significance in the analysis of the carcinoma of cheek cases.

The study of the figures given above suggests certain inferences :

The males predominate, the percentage is higher than in cheek carcinoma, the predominance being especially marked in the posterior tongue carcinomas (91·6 per cent).

There is a higher incidence in the Moslems than their general population figures would allow and higher than in carcinoma of the cheek.

The percentages in the three social strata are very different from those in cheek carcinoma. The incidence of carcinoma in the anterior two thirds of the tongue is very much higher in the lower middle class than in labour and is very

TABLE IV.—*Patients with Carcinoma of Tongue Compared with a Control Group*

| Factor studied | Carcinoma tongue Group total No. 71 Anterior 2/3 (%) | Carcinoma tongue Group total No. 71 Posterior 1/3 (%) | Control group Group No. 278 (%) |
|---|---|---|---|
| 1. Sex— | | | |
| Males | 75 | 91·6 | 50 |
| Females | 25 | 8·4 | 50 |
| 2. Religion— | | | |
| Hindus | 75·2 | 81·8 | 84·99 |
| Christians | 5·7 | 0 | 2·5 |
| Moslems | 19·1 | 18·2 | 9·93 |
| 3. Social status— | | | |
| Upper middle class | 2·88 | 23·8 | Data not available |
| Lower middle class | 60 | 52·4 | |
| Labour | 37·2 | 23·8 | |
| 4. Environment— | | | |
| Rural | 69 | 59 | 75·6 |
| Urban | 31 | 41 | 24·4 |
| 5. Diet— | | | |
| Vegetarian | 17·1 | 33 | 20 |
| Non-vegetarian | 82·9 | 67 | 80 |
| 6. Habits— | | | |
| Betel, nut and tobacco chewing | 80·5 | 33 | 12·5 |
| Betel and nut chewing | 16·6 | 29 | 51·8 |
| Smoking | 69·4 | 58·3 | 47 |
| Alcohol | 22·2 | 8·3 | Data not available |
| 7. Family history— | | | |
| Cancer in the family at any site | 2·8 | 4 | 5·7 |
| Oral cancer in the family | — | — | Not taken |
| 8. Pre-existing illness— | | | |
| Dental sepsis | 100 | 75 | 50 |
| Syphilis | 14·2 | 4 | 3·57 |
| Tubercle | 0 | 4 | 1 |
| Diabetes | 0 | 0 | 0·7 |
| Hypertension | 2·8 | 16·6 | 0·7 |
| Chronic glossitis | 28 | 8·3 | 6 |
| Virus infection | 0 | 0 | 0 |
| Anaemia | 8·3 | 4 | 1·4 |

low in the upper classes. This is almost a reversal of the position obtaining in carcinoma of the cheek where labour incidence was by far the highest. The social incidence of carcinoma of the posterior third of the tongue shows another very interesting change. The figures for the upper middle class leap from ·009 per cent in cheek cancers and 2·85 per cent in the anterior tongue cancers to 23·87 per cent in the posterior tongue cancers. Labour shows the lowest incidence and the lower middle classes the highest. Considering the general labour population in the country the incidence of tongue cancer in labour is very much lower than would be expected. The lower middle classes show a slightly but definitely higher incidence than their population trend permits. It is the upper classes who exhibit a remarkably high incidence in posterior tongue cancers when account is taken of the fact that they constitute only a microscopic minority of the population.

The posterior tongue cancers also show a higher urban incidence compared to the control group.

Diet seems of no significance nor does there appear to be any familial or hereditary tendency.

The betel and nut and tobacco chewing habit in the anterior tongue cancer patients is as high (80·5 per cent) as in the carcinomas of the cheek and significantly higher than in the control group, while in the posterior tongue cancer patients there is a sharp decline in the chewing figures to 33 per cent. The incidence of the tobacco smoking habit shows a sharp rise in both the anterior tongue cancers (69 per cent) and posterior tongue cancers (58 per cent) over the cheek cancer (26 per cent) and is also higher than in the control group. Pure betel and nut chewing appears to be of no significance nor the alcohol habit.

The incidence of dental sepsis in anterior tongue cancers is 100 per cent and in posterior tongue cancers 75 per cent.

There does not seem to be any other factor of significance in pre-existing local pathology.

### DISCUSSION

The foregoing study, and more convincing still the histories that are daily elicited in our outpatient department, have convinced us all that the habit of prolonged combined tobacco, betel and nut chewing over a long period (15–20 years) together with the oral sepsis thus engendered is the most important single factor in the causation of oral squamous cell carcinomas. Sex is of no importance and follows only the trend of the chewing habit. Over 90 per cent of our patients and our controls suffered from some degree of clinically obvious vitamin A and vitamin B deficiency. There was no clinical evidence of vitamin C and D deficiency. Anaemia did not seem to be of much significance. The social incidence also followed only the habit trends. The maximum tobacco, betel and nut chewers were in the lower middle classes and labour. The upper classes rarely chew tobacco. Oral hygiene amongst labour is totally lacking while it is very much better in the lower middle class. This may explain the lower incidence of carcinoma of the cheek in the lower middle classes than in the labour. Another interesting fact that we noticed generally was that the variation in cheek carcinoma incidence from one region to another of our and neighbouring states followed the tobacco, betel and nut chewing habit rather closely. We would have very much liked to study more carefully this aspect of geographical pathology had our finances permitted it. Another interesting fact was that the cheek carcinomas usually occurred on the side against which the chewer held the tobacco bolus. The chronic chewer chews a compound of dried and cured tobacco leaf, powdered betel nut and betel leaves with a touch of moistened and powdered lime. He intermittently chews this material and then holds the chewed bolus against one or the other cheek in the alveolo-labial sulcus for a while and then chews it again. This process goes on continuously for a few hours and is then replaced by a fresh chew. It is virtually chain chewing. The father passes on the habit to the son and all our cancer patients have been chewers for not less than 15–20 years, commencing usually at the age of 20 when they enter the father's profession. Most chewers have the habit of holding the bolus against one particular cheek.

Tobacco, betel and nut chewing and sepsis seem to have a very definite relationship to squamous cell carcinoma of the anterior two thirds of the tongue. The relationship follows very closely that found in cheek carcinomas. One additional factor has, however, also to be considered and that is tobacco smoking.

69 per cent of anterior tongue cancer patients were smokers compared to 26 per cent of cheek cancer patients and 47 per cent in the control group. Over 80 per cent of our patients were cheroot or bidi smokers. A beedi is just a roll of specially prepared dried tobacco leaves tied with a thread. There is no enclosing paper as in a cigarette. It is about an inch and a half long and expanded at one end. It is smoked at the narrower end. It is possible that the inhaled tobacco smoke makes its own carcinogenic contribution.

Tobacco smoking appears to assume a more important role in carcinoma of the posterior third of the tongue. Tobbacco, betel and nut chewers in posterior tongue cancers were only 33 per cent compared to 85 per cent in cancers of the cheek and 80 per cent in anterior tongue cancers, but tobacco smokers were 58·3 per cent. However, the smokers in the control were 47 per cent. There is one more significant fact and that is the comparative high incidence of posterior tongue carcinomas in the upper classes of the social strata. Could this be due to the comparatively greater prevalence of smoking and heavier smoking in that opulent class? But it is reasonable to believe that a substance that is definitely carcinogenic in the oral cavity could contribute to carcinogenesis elsewhere.

The greater frequency of posterior tongue cancer in Bombay, in the urban population in Madras State and in Moslems and in males compared to the control figures also suggests the carcinogenic role of tobacco smoking. It is well known that the smoking habit is much more common in the urban population and in Moslems in Madras State than in rural areas or other communities. Women, too, very rarely smoke.

It is our conviction that adequate vitaminisation may to a certain extent act as a protective factor against the epithelial alterations that finally summate into malignancy. Avitaminosis in itself does not, however, contribute to carcinogenesis.

(Note : Only those who smoked 10 beedis a day for over 10 years were considered regular smokers.)

### SUMMARY

Cancer of the mouth and pharynx constituted 48 per cent of all malignancies seen at the Cancer Institute, Madras, of which 71 per cent were of the buccal mucosa and 22 per cent of the lingual mucosa. 347 cases of squamous cell carcinoma of the mouth were analysed for possible aetiological factors. Sex, religion, environment, diet, pre-existing illness like anaemia, syphilis, tuberculosis, diabetes, hypertension and virus diseases were not of significance. There was no relationship between achlorhydria and oral cancer. The habit of tobacco and betel and nut chewing appeared most significant. The carcinogenic action of these seem to be promoted by dental sepsis.

In carcinomas of the anterior two thirds of the tongue, the same aetiological factor held good though tobacco smoking apparently played a dominant role in carcinoma of the posterior third of the tongue.

### REFERENCES

KHANOLKAR, V. R.—(1958) ‘ Cancer ’ Edited by Ronald Raven. London (Butterworths) Vol. 3, p. 272.—(1944) *Cancer Res.* **4**, 313.

SANGHVI, L. D., RAO, K. C. M. AND KHANOLKAR, V. R.—(1955) *Brit. med. J.*, **1**, 1111.